# Open questions on bonding involving lanthanide atoms

**DOI:** 10.1038/s42004-022-00630-6

**Published:** 2022-02-02

**Authors:** T. Vitova, P. W. Roesky, S. Dehnen

**Affiliations:** 1 Institute for Nuclear Waste Disposal (INE), Karlsruhe Institute of Technology, P.O. 3640, D-76021 Karlsruhe, Germany; 2 Institute for Inorganic Chemistry, Karlsruhe Institute of Technology, P.O. 3640, D-76021 Karlsruhe, Germany; 3 Fachbereich Chemie and Wissenschaftliches Zentrum für Materialwissenschaften, Philipps-Universität Marburg, D-35043 Marburg, Germany

**Keywords:** Coordination chemistry, Chemical bonding, Optical materials

## Abstract

In-depth understanding of the bonding characteristics of the lanthanide ions in contemporary lanthanide-based materials is mandatory for tailoring their properties for novel applications. Here, the authors elaborate on open questions regarding the bonding situation in mainly molecular lanthanide (4f) compounds, where, as compared to their actinide (5f) analogs in which covalency of the bonds is a common feature, this is still under discussion for the 4f compounds.

The bonding properties of the lanthanide 4f elements (Ln = La–Lu), and the electron-poor actinide 5f elements (An = Th–Cm) exhibit similarities in some regards, but generally, they show fundamental differences. Formally, the oxidation states span a range from +II to +IV for the lanthanides, whereas they vary from +II to +VIII for actinides. In nature, commonly found states are +III (Ln) or +IV/+VI (An). The general notion is that the lanthanides form predominantly ionic bonds, at least in their +III oxidation state, whereas the actinides are capable of undergoing covalent bonding, but to a lesser extent than the d-block transition metals. This again strongly depends on the oxidation state of the actinides, with the most prominent example being the covalent bonds formed in the actinyl ions [An(V)/(VI)O_2_]^1+/2+^. However, this traditional view on the chemical behavior of the elements at the bottom of the periodic table is rapidly changing nowadays. Advances in spectroscopy give indications that lanthanide atoms can also be involved in covalent bonding interactions. This opens a question about the nature of such bonds, which will also affect further chemical and physical properties like reactivity, bond stability, and emission as well as magnetic behavior. Advances in Ln(II) and Ln–(inter)metallic coordination chemistry unveil new lanthanide-based materials with unexplored physical and chemical properties—a treasure chest for the development of novel applications with tailored properties.

## Covalency in lanthanide bonding

**Fig. 1:**
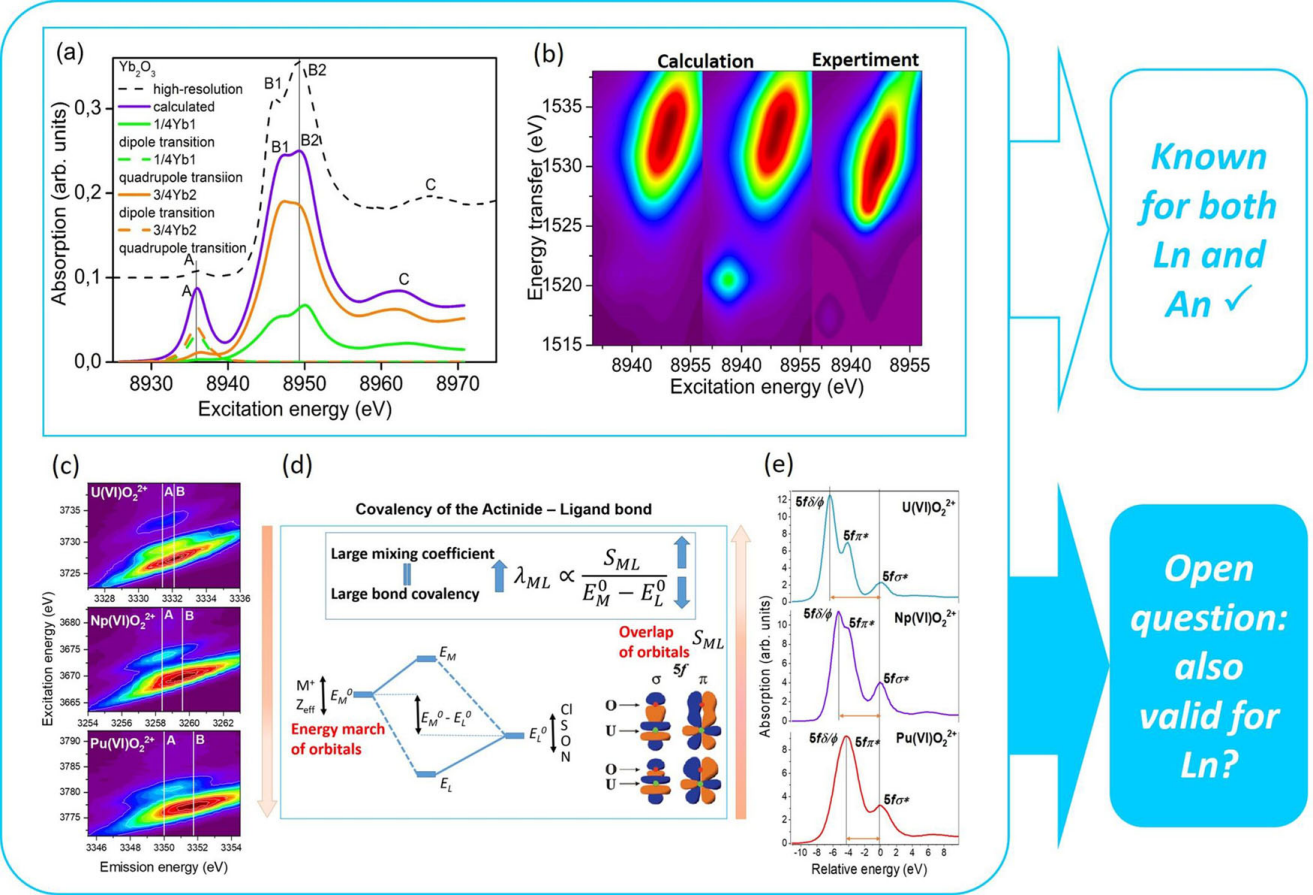
Spectroscopic methods probing in detail the bonding properties of lanthanide and actinide atoms in corresponding compounds. **a** Yb L_3_-edge HR-XANES of Yb_2_O_3_ and **b** Yb L_3_ 3d4f CC-RIXS experiment and calculations of Yb_2_O_3_. **c** The RIXS maps of UO_2_^2+^, NpO_2_^2+^, and PuO_2_^2+^ in aqueous solution. The white lines mark the maximum intensity of the normal emission (line A) and resonant emission (line B). The shift between lines A and B grows from top to bottom (pink arrow) and correlates with the increasing localization of the 5f valence orbitals from U to Pu (E_M_^0^ shifts down) and thus the energy match of orbitals improves. **d** The dependency of the mixing coefficient (*λ*_*ML*_) on the energy match E_M_ − E_L_ (energy difference) and the overlap of the metal and ligand valence orbitals S_ML_, the molecular orbital (MO) scheme depicts how the energy match changes as a function of Z_eff_/M^+^ oxidation state^1^. The overlap of M and L valence orbitals for two U–O bond distances. **e** The U M_4_ edge HR-XANES spectra of UO_2_^2+^, NpO_2_^2+^, and PuO_2_^2+^. The energy shift between the 5f﻿δ/ϕ and 5fσ* peaks rises from bottom to top (pink arrow) and thus the orbital overlap increases from Pu to U. Panels **a** and **b** are adapted from ref. ^17^, **c** and **e** from ref. ^13^.

The classical definition for a covalent chemical bond is the accumulation of electron density between the involved atoms. The covalency of the bond can be described by the mixing coefficient, and it will increase if the orbital overlap is large or the energy difference between metal and ligand orbitals is minimized (cf. Fig. 1d)^1^. The interconnection between the orbital overlap and energy-degeneracy-driven covalency, and the subsequent impact on reactivity or bond stability for both lanthanide and actinide compounds, is not well understood and at the forefront of lanthanide and actinide research. Recently, the group of Gregory Nocton reviewed Yb, Eu, Tm, and Ce organometallic lanthanide compounds with intermediate oxidation states like for example LnCp_3_ (Ln = Ce, Eu, or Yb; Cp = cyclopentadienide, (C_5_H_5_)^−^), [Cp*_2_Yb(bipy)], [Cp*_2_Yb(dad)], and [Cp*_2_Yb(phen)] (Cp* = pentamethylcyclopentadienide, (C_5_Me_5_)^−^; bipy = 2,2'-bipyridine, C_10_H_8_N_2_; dad = 1,4-diazabutadiene; phen = phenanthroline, C_12_H_8_N_2_)^2^. These materials exhibit charge transfer from the ligand to the metal, indicative of increased bond covalency as a result of improved energy match between the ligand and the lanthanide metal. Generally, low ionization energies of the respective lanthanide ion allow for a higher covalency of the compound. Given its low ionization energy, this may be one reason why most of the recent studies into lanthanide covalency of the trivalent ions are dealing with Ce compounds^3–9^.

Although the formula of cerocene, [Ce(cot)_2_] (cot = cyclooctatetraene dianion, (C_8_H_8_)^2^^−^, for instance, would suggest a +IV oxidation state, it was demonstrated, e.g., by Ce K-edge X-ray absorption near edge structure (XANES) to accord better with a +III state^3,4^. Recently, Stefan G. Minasian, S. Chantal E. Stieber et al. clearly showed that the Ce atom in [Ce(cot)_2_] forms covalent bonds with the participation of Ce 4f electrons by C K-edge XANES, Ce M_4,5_-edge XANES, and Configuration Interaction (CI) computations^5^. These unusual bonding properties of the Ce atom lead to quantum chemical phenomena rare for molecular systems. The authors discuss evidence that the overlap-driven covalency is more important for the stabilization of the chemical bond in [U(cot)_2_] than in [Ce(cot)_2_], even though the mixing coefficient is comparable. The latter results from the better energy match of the 4f orbital at a Ce^3+^ ion as compared to the 5f orbital at a U^3+^ ion with the ligand orbitals. Lukens et al. determined the stabilization of the ground state of [Ce(cot)_2_], which is a result of mixing between the ligands’ orbitals and the 4f orbitals of the Ce atom using the Hubbard molecule model^6^.

Significant stabilization of Ce(IV) was observed through the tris(piperidinyl)imidophosphorane ligand, [NP(pip)_3_]^−^^7^. Spectroscopic studies (UV-visible, electron paramagnetic resonance, and Ce L_3_-edge X-ray absorption spectroscopies), in conjunction with density functional theory studies, of the homoleptic imidophosophorane redox pair [Ce(NP(pip)_3_)_4_] and [(Et_2_O)KCe(NP(pip)_3_)_4_] reveal dominant covalent metal–ligand interactions. The electronic basis of the destabilization of the f orbitals in these complexes was interrogated by Ce L_3_-edge XANES and theoretical modeling.

In comparison to the actinides, Eric J. Schelter et al. provided experimental evidence for the larger covalent character of 4f^0^5d^0^ Ce(IV) multiple bonds as compared to its 5f^0^6d^0^ Th(IV) actinide congener by comparing a series of Th(IV) and Ce(IV) imido complexes^8^. This is in line with the recent literature dealing with the comparison of the covalent character of tetravalent cerium and the tetravalent actinides^9,10^.

Apart from Ce, there are examples for Ln(IV)O_2_ (Ln = Ce, Pr, and Tb) illustrating by O K-edge XANES and Ln L_3_/M_4,5_-edge XANES that Ln 4f, 5d and O 2p orbitals mix, i.e., increasing bond covalency, as a result of good energy match between Ln and ligand orbitals^11^. In addition, in a series of [Ln(III)Cl_6_]^3–^ complexes (Ln = Ce, Nd, Sm, Eu, and Gd) and [Ce(IV)Cl_6_]^2–^, Cl K-edge XANES spectroscopy and density functional theory (DTF)/time-dependent DFT (TD-DFT) computations revealed the participation of Ln 5d orbitals in the bonding for all molecules, whereas also Ce 4f was mixed with ligand orbitals for [Ce(IV)Cl_6_]^2–^ ^12^.

In all cases where an increase of mixing coefficient/intermediate valence state is observed, the Ln 5d orbitals play a substantial role. The many examples in the literature show that actually the Ln 5d orbitals are rarely completely empty as the tabulated ground state electronic configurations for most of the lanthanide elements state. Since they also have larger spatial extension compared to the 4f orbitals, is it possible that orbital overlap between metal and ligand valence orbitals, not only energy match, also play a role in the covalency of the Ln–ligand bonds?

Recently, we demonstrated that the An M_4,5_-edge high energy resolution XANES (HR-XANES) and resonant inelastic X-ray scattering (RIXS) spectroscopy techniques can be used to distinguish between the classical notion of overlap-driven covalency and energy-degeneracy-driven covalency for actinyl compounds (cf. Fig. 1c, d and e)^13^. It would be beneficial to develop and apply similar approaches for Ln-based compounds. A well-established way to probe the bond covalency, and thus the overall bonding properties, is to evaluate the level of mixing of metal and ligand valence orbitals (the mixing coefficient in Fig. 1d), through ligand K-edge XANES spectra^5,11,12^. The orbital overlap of metal and ligand orbitals and the energy degeneracy can be explored by valence band X-ray emission spectroscopy selective to Ln 5d or 4f electronic density in the valence band. Specifically, probing the occupied Ln 4f orbital is of interest to answer the open question if lanthanide atoms can form covalent bonds using their 4f orbitals similarly to actinide atoms, for which the usage of 5f orbitals has been documented. These studies have recently become possible due to experimental advances at the European Synchrotron Radiation Facility (ESRF), allowing energy resolution of 30 meV (valence band (VB)-HR-RIXS at the Ln M_4,5_-edges)^14^.

## Unusual Ln(II)-based and Ln–(inter)metallic cluster-based materials

Unusual Ln(II)-based molecular and Ln–(inter)metallic cluster-based compounds have largely unexplored bonding properties, and it is unclear whether and how commonly the lanthanide atoms in those materials can form covalent bonds. Ln(II) materials outside of those based on Sm(II), Eu(II), and Yb(II) are rare, but the group of William J. Evans synthesized, e.g., [K(2.2.2-cryptand)][Ln(II)(C_5_H_4_SiMe_3_)_3_] (2.2.2-cryptand = 4,7,13,16,21,24-hexaoxa-1,10-diazabicyclo[8.8.8]hexacosane, C_18_N_2_H_36_O_6_) across almost the entire Ln series (excluding Pm)^15^. The groups of Enrique Batista, Stosh A. Kozimor and Ping Yang characterized the Ln(II) oxidation states using classical Ln L_3_-edge XANES spectroscopy and DFT as well as complete active-space second-order perturbation theory (CASPT2) computations^15^. This conventional experimental method is powerful; however, in case the additional electron is transferred to the Ln 5d instead of the 4f orbitals or activation of Ln(II)/Ln(III) 4f^n^5d^0^ to Ln(II)/Ln(III) 4f^n-1^5d^1^ takes place, the more advanced Ln L_2,3_-edge HR-XANES spectroscopy would be much more instructive. We and Kristina O. Kvashnina, Pieter Glatzel et al. showed that the method resolves the transitions to 4f and 5d states for Ln(III) materials and thus allows their separate evaluation upon bond variations^16^ (cf. Fig. 1a, b)^17,18^. Figure 1a, b shows that the pre-edge probes the energy position of the 4f orbitals and gains intensity when Ln 4f are mixed with 5d orbitals in Yb_2_O_3_. This example for a solid-state compound demonstrates the potential of the spectroscopic technique for probing the bonding properties of Ln molecular materials. The Ln/An L_3_-edge HR-XANES spectrum measures the energy shift between 4f/5f and 5d/6d orbitals, and this spectroscopic tool is available for Ln (4f, 5d) and An (5f, 6d)^17,19^. From the Ln 4f with 5d mixing and the energy shift, we can learn to what extent electronic density is transferred from the 4f to the Ln 5d orbitals, and we can use the findings to evaluate these orbitals’ involvement in the Ln–ligand bonding. Similar questions regarding the involvement of lanthanide orbitals in covalent bonding arise when studying intermetalloid cluster compounds, in which Ln^n+^ ions are embedded in a shell of (semi)metal atoms^20,21^, in comparison with An-centered analogues^22,23^. We raise the question—can we develop spectroscopic tools to measure also the two parameters to which is proportional the mixing coefficient *λ*_*ML*_, energy match (E_M_ − E_L_) and orbital overlap (S_ML_), in order to study their interconnection with bond stability and reactivity also for the lanthanide elements (Fig. 1d)?

## Outlook

The recent studies mentioned above showcase both the possibility and also the necessity to dig more deeply into the nature of the lanthanide–element bond. The paradigm of being mostly ionic has dominated the discussion and also the design of new lanthanide compounds and Ln-based materials, but a new and modern view of the bonding characteristic will allow for a rapid and sustainable change. Further developments of analytical techniques have allowed for new and fresh views into lanthanide compounds, and these have set the course for future work. It has been discussed that HR-XANES and RIXS are powerful spectroscopic tools for the investigation of bonding character. This way, the open question about the nature of the bonds involving Ln atoms may be answered. We envisage that once the bonding is much better understood, the design of compounds that exhibit new physical and chemical properties for applications like activation of small molecules, materials with unusual reactivity, luminescent materials, quantum materials, or single molecular magnets with high blocking temperature will emerge, which represents both a great challenge and a fantastic opportunity.
